# Supplementary figures and images for: Synchrony on the reef: how environmental factors shape coral spawning patterns in Acropora corals in the Maldives
Source: PeerJ. 2025 May 28;13:e19447. doi: 10.7717/peerj.19447 (PMC12126091; doi:10.7717/peerj.19447)

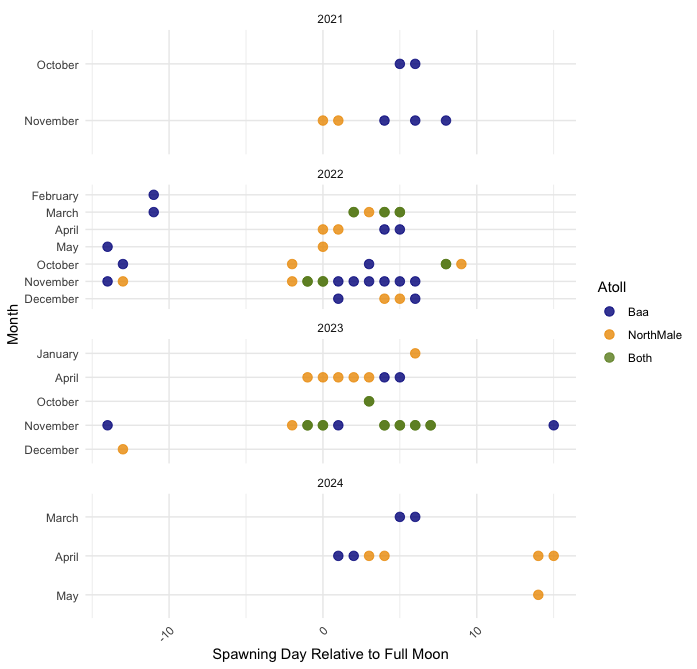

Supplement: Supplemental Information 1 — On the x-axis, 0 corresponds to the Full Moon, and 15 corresponds to the New Moon. This figure shows the months in which mature gametes were identified and thus night spawning surveys were conducted. Further evident is Acropora corals protracted breeding in the Maldives season and records of spawning across multiple lunar phases. [file peerj-13-19447-s001.png]

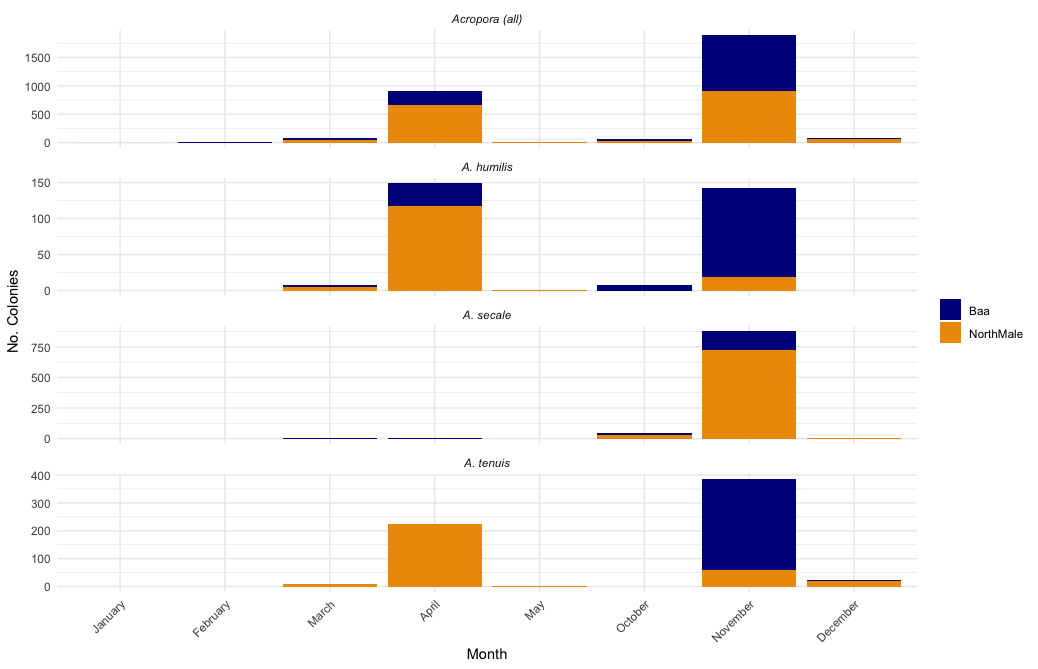

Supplement: Supplemental Information 2 — The peak spawning months of the Maldives for Acropora corals: April and November. [file peerj-13-19447-s002.png]

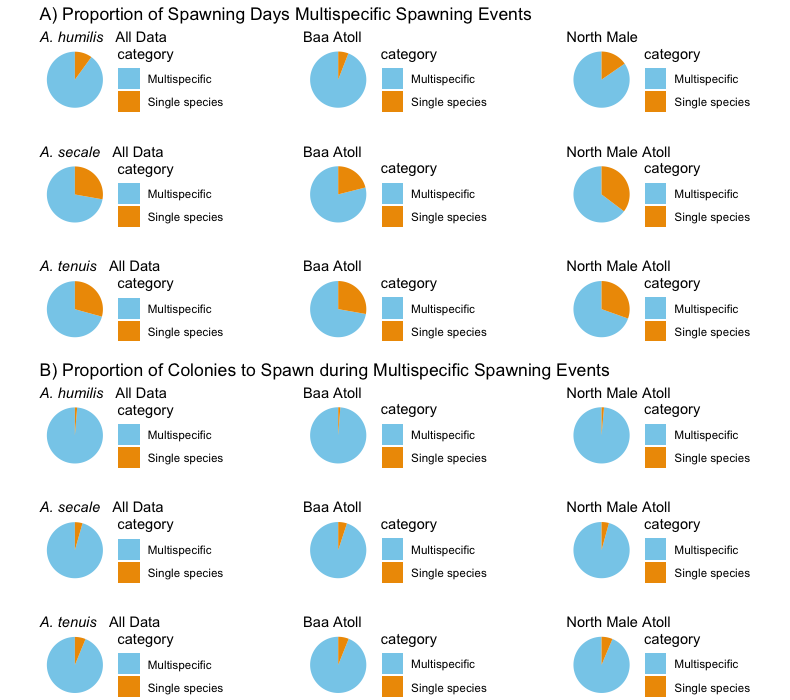

Supplement: Supplemental Information 3 — (A) Proportion of spawning days when A. humilis, A. secale, or A. tenuis participated in multispecific spawning events versus spawning alone. (B) Proportion of colonies of A. humilis, A. secale, or A. tenuis to spawn during multispecific spawning events versus spawning alone. For both (A) and (B), data is displayed for both atolls combined and individually for each. Only days in which the mentioned species spawned were included. [file peerj-13-19447-s003.png]
